# Effect of Ischemic Postconditioning and Atorvastatin in the Prevention of Remote Lung Reperfusion Injury

**DOI:** 10.21470/1678-9741-2017-0022

**Published:** 2018

**Authors:** Carlos Henrique Marques dos Santos, Doroty Mesquita Dourado, Baldomero Antonio Kato da Silva, Henrique Budib Dorsa Pontes, Euler de Azevedo Neto, Giovanna Serra da Cruz Vendas, Ian de Oliveira Chaves, João Victor Cunha Miranda, João Victor Durães Gomes Oliva, Letícia do Espírito Santo Dias, Murillo Henrique Martins de Almeida, Trícia Luna Sampaio

**Affiliations:** 1 Universidade Anhanguera (Uniderp), Campo Grande, MS, Brazil.; 2 Universidade Federal do Piauí (UFPI), Teresina, PI, Brazil.

**Keywords:** Ischemia, Reperfusion Injury, Ischemic Postconditioning, Hydroxymethylglutaryl-CoA Reductase Inhibitors, Lung

## Abstract

**Objective:**

The aim of the present study was to evaluate the ability of ischemic postconditioning, atorvastatin and both associated to prevent or minimize reperfusion injury in the lung of rats subjected to ischemia and reperfusion by abdominal aortic clamping.

**Methods:**

We used 41 Wistar norvegic rats, which were distributed into 5 groups: ischemia and reperfusion (I/R), ischemic postcondictioning (IPC), postconditioning + atorvastatin (IPC+A), atorvastatin (A) and SHAM. It was performed a medium laparotomy, dissection and isolation of the infra-renal abdominal aorta; except for the SHAM group, all the others were submitted to the aortic clamping for 70 minutes (ischemia) and posterior clamp removal (reperfusion, 70 minutes). In the IPC and IPC+A groups, postconditioning was performed between the ischemia and reperfusion phases by four cycles of reperfusion and ischemia lasting 30 seconds each. In the IPC+A and A groups, preceding the surgical procedure, administration of 3.4 mg/day of atorvastatin was performed for seven days by gavage. After the surgical procedure, the right caudal lobe was removed from the lung for histological study, using tissue injury score ranging from grade 1 (normal tissue) to grade 4 (intense lesion).

**Results:**

The mean lung injury was 3.6 in the I/R group, 1.6 in the IPC group, 1.2 in the IPC+A group, 1.2 in the A group, and 1 in the SHAM group (*P*<0.01).

**Conclusion:**

Ischemic postconditioning and atorvastatin were able to minimize lung reperfusion injury, alone or in combination.

**Table:** 

Abbreviations, acronyms & symbols	
A	= Atorvastatin group
IPC	= Ischemic postcondictioning
IPC+A	= Postconditioning + atorvastatin group
IR	= Ischemia and reperfusion
I/R	= Ischemia and reperfusion group
ROS	= Reactive oxygen species
TNF	= Tumor necrosis factor

## INTRODUCTION

Reperfusion is a fundamental step in the treatment of ischemia. However, clinical and experimental evidence shows that the main events leading to cell and tissue dysfunction are related to reperfusion^[1]^.

The ischemia and reperfusion (IR) injury constitutes a pathophysiological event common to several diseases of daily clinical practice. The lung can be the target of the IR lesion directly, as in pulmonary edema after transplantation or in the resolution of thromboembolism, or be reached at a distance, as in cases of shock or reperfusion injury in the intestine or lower limbs, as occurs in the aortic clamping, used in aneurysm surgeries^[2]^.

It was believed that the lung was more resistant to ischemic lesions than other organs. Two factors contributed to this: the presence of bronchial circulation in addition to the pulmonary circulation and the fact that interruption of pulmonary blood flow is not accompanied by hypoxia since alveolar ventilation is maintained. The lung can be considered the only organ that can suffer ischemia without hypoxia^[3]^.

However, evidence has emerged in recent years that the lung may not be completely immune to reperfusion injury, despite maintained gas exchange, since reactive oxygen species (ROS) act systemically. In surgeries with temporary aortic occlusion, pulmonary edema is a frequent complication, with a multifactorial etiology, including reperfusion injury. Already during ischemia, there is an increase in pulmonary arterial pressure, a factor that may favor the formation of edema in the lungs. This increased resistance in the pulmonary circulation results in part from a greater blood flow due to its redistribution to the territory above the occlusion and from the increase in left ventricular end-diastolic volume, whose emptying is impaired by the increase that the occlusion of the aorta imposes on post-loading^[4]^.

IR is associated with the production of another inflammatory mediator, tumor necrosis factor (TNF). Lesion of the intestinal mucosa by IR allows the release of endotoxin into the portal circulation, inducing the production of TNF by liver macrophages. Increased TNF in the systemic circulation is capable of leading to inflammatory lung injury, characterized by accumulation of neutrophils. This sequence of events was demonstrated by Caty et al.^[5]^ in an IR model by temporary occlusion of the superior mesenteric artery in rats. After reperfusion, endotoxin levels in portal venous blood and TNF were increased in the systemic circulation. In parallel, there was accumulation of neutrophils in the lungs and increased pulmonary capillary permeability.

Some techniques for protection against reperfusion injury have already been tried and tested, and among them, ischemic postconditioning (IPC), which consists of one or more short cycles of reperfusion, followed by one or more short cycles of ischemia, immediately after the ischemic phase and before permanent reperfusion occurs. Although IPC has already shown a protective effect in many organs submitted to IR as well as in distance protection^[6]^, its efficacy in the prevention of remote lung injury is still very early^[7]^.

Much has been studied about the pathophysiology of reperfusion injury and some mechanisms have already been well evidenced, such as the role of free radicals, vascular endothelial dysfunction, and neutrophil-mediated injury^[1]^. Recently, there has been an increase in interest in statins, drugs known for their antidislipidemic effect, this time due to its pleiotropic effect, which is characterized by anti-inflammatory properties, immunomodulating, antithrombogenic actions and improvement of endothelial function^[8]^. Recent experimental studies^[9]^ have shown promising results with the use of statins demonstrating their role in the protection against IR injury, a fact that led us to inquire about their benefits facing reperfusion injury.

The aim of the present study was to evaluate the effect of IPC and atorvastatin, alone and in combination, in the prevention of reperfusion injury in the lungs of rats subjected to IR by aortic clamping.

## METHODS

The study was approved by the Committee of Ethics in Animal Experimentation of the Universidade Anhanguera-Uniderp. A total of 41 Wistar norvergic male rats weighing 250 g to 300 g were collected from the Hospital Veterinário da Universidade Anhanguera-Uniderp. The animals were kept in cages at ambient temperature of approximately 23ºC with 12h light cycles and received water and food *ad libitum.*

The animals were distributed in the following groups:

- Ischemia and Reperfusion group (I/R): nine rats were submitted to ischemia for 70 minutes by aortic clamping, followed by reperfusion of 70 minutes;
- Ischemic Postconditioning group (IPC): nine rats were submitted to the ischemia procedure for 70 minutes by aortic clamping and reperfusion for 70 minutes. Between ischemia and reperfusion, four cycles of reperfusion (30 seconds each) were performed, interspersed by four cycles of ischemia (30 seconds each);
- Ischemic Postconditioning + Atorvastatin group (IPC+A): nine rats received 3.4 mg/day of atorvastatin, one dose per day through the gavage method, for seven days and then were submitted to the ischemia procedure for 70 minutes by aortic clamping and reperfusion for 70 minutes. Between ischemia and reperfusion, four cycles of reperfusion (30 seconds each) were performed, interspersed by four cycles of ischemia (30 seconds each);
- Atorvastatin group (A): nine rats received 3.4 mg/day of atorvastatin, one dose per day through the gavage method, for seven days, and then were subjected to the ischemia procedure for 70 minutes by aortic clamping and reperfusion for 70 minutes;
- SHAM group: five rats submitted to laparotomy, dissection and isolation of infra-renal aorta.

The animals were anesthetized by intraperitoneal injection of a 2:1 solution of ketamine hydrochloride (Cetamin^®^), 50 mg/mL, and xylasine hydrochloride (Xilazin^®^), 20 mg/mL, respectively, at a dose of 0.1 mL/100 g.

After anesthesia, the rats were submitted to a median longitudinal laparotomy of approximately four centimeters, exteriorization of the small intestine, identification and dissection of infra-renal abdominal aorta artery.

In all groups except SHAM, the abdominal aorta was occluded by atraumatic vascular clamp that remained for 70 minutes (ischemia phase). After clamp placement, the small intestine was repositioned into the abdominal cavity and the surgical wound was closed with continuous suture of the skin with 4-0 monofilament nylon. After the ischemia phase, the abdominal wall was reopened by removal of the suture and in the I/R and A groups, the vascular clamp was removed, initiating the reperfusion phase, lasting 70 minutes. In the IPC and IPC+A groups, preceding the reperfusion phase, the IPC was performed by four cycles of reperfusion (removal of the atraumatic vascular clamping of the abdominal aorta) with duration of 30 seconds each, interspersed by four cycles of ischemia (occlusion of the abdominal aorta artery by atraumatic vascular clamp), also with duration of 30 seconds each.

In all groups after the beginning of the reperfusion phase, the abdomen was closed again by continuous suturing of the skin with 4-0 monofilament nylon thread until the end of the experiment.

In the SHAM group, only a median longitudinal laparotomy of approximately four centimeters was performed, with exteriorization of the small intestine, identification and dissection of the infra-renal abdominal aorta artery, remaining anesthetized for 140 minutes.

After the reperfusion phase, all animals were submitted to median thoracotomy and resection of the right caudal lung lobe, and these specimens were washed with saline solution and placed in 10% formaldehyde solution for histological analysis.

Euthanasia was performed by intraperitoneal administration of a lethal dose of ketamine + xylazine hydrochloride (0.4 mL/100 g).

The slides were prepared with the harvested material, which was stained with hematoxylin-eosin and analyzed by optical microscopy by a single observer, without prior knowledge of it on the group belonging to each rat.

Pulmonary segments were classified according to the degree of tissue injury as stated by Greca et al.^[10]^.

- Grade 1 (normal): normal parenchyma under optical microscopy;
- Grade 2 (mild): focal edema in few alveolar septa, mild congestion, neutrophils in alveolar septa, less than 50 per large increase field;
- Grade 3 (moderate): moderate edema in alveolar septa or mild edema in several septa, moderate congestion, neutrophils in alveolar septa between 50 and 100 per large increase field;
- Grade 4 (intense): severe edema in alveolar septa or mild edema in several septa, moderate congestion, neutrophils in alveolar septa, more than 100 per field.

Measurements were expressed as mean and standard deviation in the variables whose distribution was normal, and in the median and interquartile range when normality was not observed. For the verification of normality, the Shapiro-Wilk test was used. For the intergroup comparison, Kruskal-Wallis test with Dunn's *post hoc* test was used. Significance level was considered *P*<0.05.

## RESULTS

**Fig. 1:**
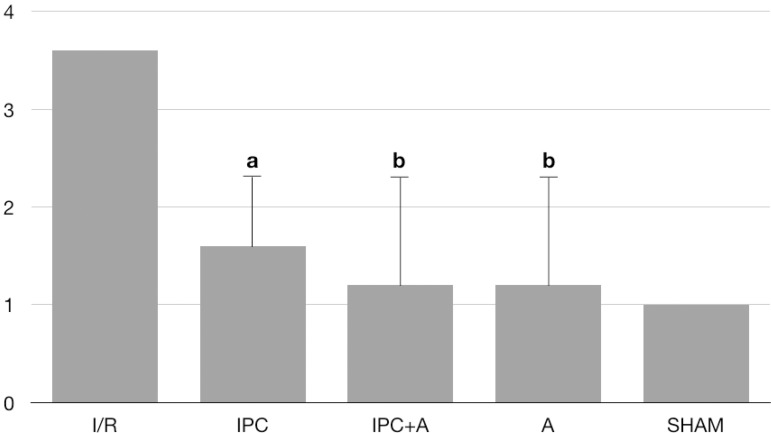
Comparison of the medians of the degrees of lung injury among the different groups analyzed (Kruskal-Wallis P=0.0029; "a" P<0.01 in relation to I/R group; "b" P<0.001 in relation to I/R group).

The averages of degrees of tissue injury were 3.6 in the I/R group, 1.6 in the IPC group, 1.2 in the IPC+A and A groups, and 1 in the SHAM group (Table 1 and Figure 1).

**Table 1:** Degree of histopathological lesion in the lung parenchyma in rats per group.

Groups					
Rats	I/R	IPC	IPC+A	A	SHAM
1	4	1	1	1	1
2	4	1	1	1	1
3	4	2	2	2	1
4	4	1	1	2	1
5	4	3	1	1	1
6	3	2	2	1	
7	2	1	1	1	
8	4	1	1	1	
9	4	2	1	1	
Average	3.6	1.6	1.2	1.2	1

## DISCUSSION

Ischemia followed by reperfusion may induce apoptosis and an inflammatory response that affects tissue repair, especially in the lung. As a result, many evaluated the impact of IPC in subsequent apoptotic and inflammatory responses. In experimental IR models in rats with 30 minutes of ischemia and three hours of reperfusion there was a significant decrease in tissue necrosis with IPC. There is also a decrease in ROS generation and protection of mitochondrial integrity, suggesting that the protective effect of IPC may be the result of a reduction in the inflammatory response. However, few studies have directly assessed the impact of IPC on inflammation. IPC may limit the expression of P-selectin, which is required for neutrophil bearing and recruitment. In addition, it may reduce the accumulation of neutrophils in the affected region, decrease to ischemic vascular endothelial adhesion, and attenuate the endothelial dysfunction of the involved vessel, events that normally occur in IR^[11]^.

**Fig. 2:**
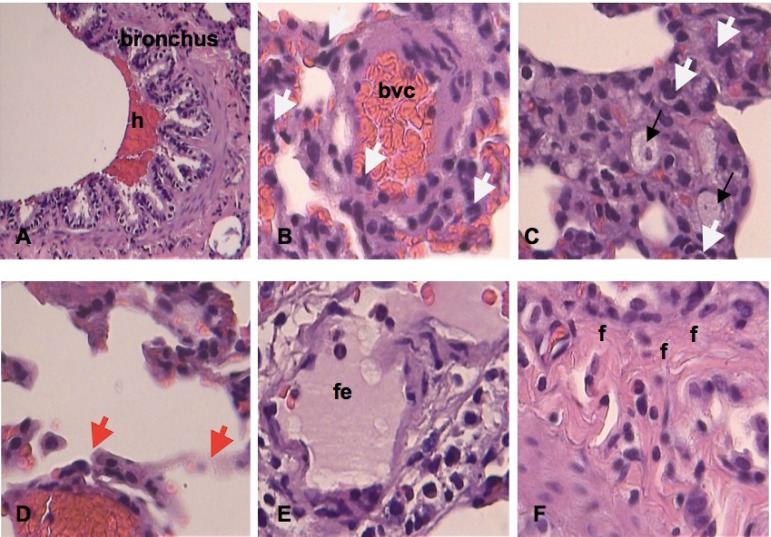
Photomicrographs of histological changes of the pulmonary parenchyma of the I/R group considering the classification of Greca et al.^[10]^. Major changes are observed predominantly, grade 4: haemorrhage (h); blood vessel congestion (bvc); cell necrosis (black arrow); neutrophils (white arrow); alveolar wall (red arrow); focal edema (fe); fibrosis (f ). HE, 10x and 40x.

**Fig. 3:**
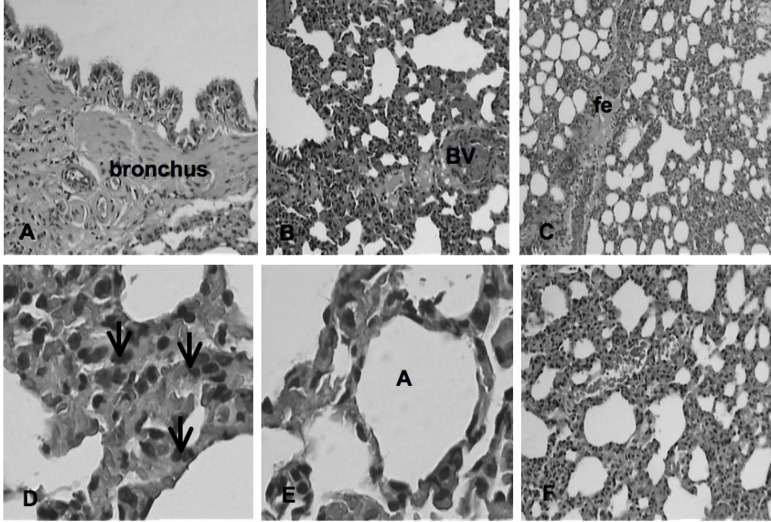
Photomicrographs of the pulmonary parenchyma of the IPC group according to Greca et al.^[10]^. It is observed: bronchus; blood vessel (BV); focal edema (fe); neutrophil (arrow); alveolus (A). HE, 10x and 40x.

**Fig. 4:**
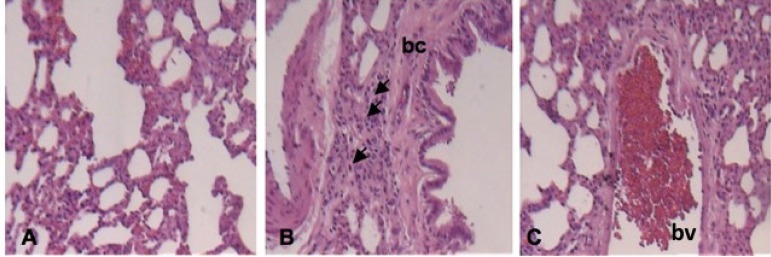
Photomicrographs of the pulmonary parenchyma of the IPC+A group according to Greca et al.^[10]^. Bronchiolus (bc); blood vessel (bv); inflammation (arrow). HE, 10x and 40x.

**Fig. 5:**
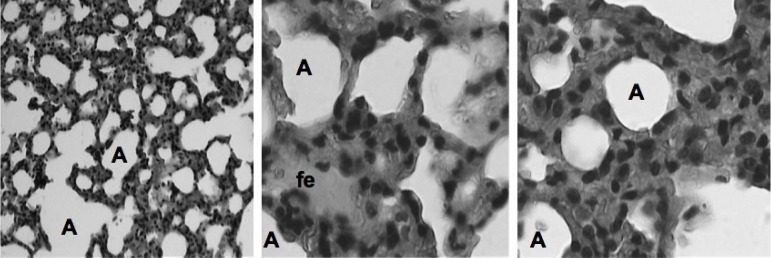
Photomicrographs of the pulmonary parenchyma of group A according to the classification of Greca et al.^[10]^. Focal edema (fe); alveolus (A). HE, 10x and 40x.

**Fig. 6:**
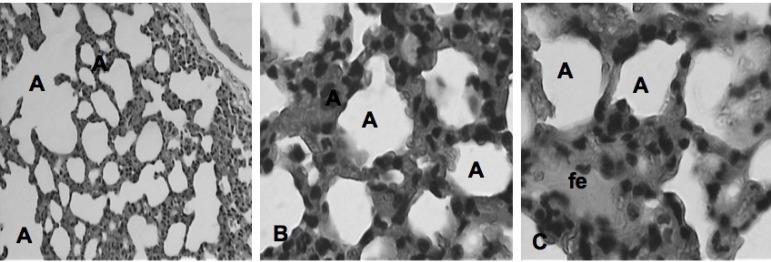
Photomicrographs of the pulmonary parenchyma of the SHAM group according to Greca et al.^[10]^. Focal edema (fe); alveolus (A). HE, 10x and 40x.

The main histological findings observed in the different groups can be observed in Figures 2 to 6.

In the present study, we observed pulmonary protection with IPC, demonstrating the efficacy of the method against this IR model, which can be justified by the fact that ROS, regardless of where they are produced, when reperfusion occurs throughout the body causing the remote reperfusion injury, so much so that in the I/R group a marked lesion in the lungs was observed. By acting as a moderator of ROS production, IPC causes less local and distant injury^[4,7]^.

The study of the remote reperfusion injury in the lungs is a relatively new subject, therefore, without publications that allow us to compare further with the present results. Dorsa et al.^[7]^ also performed ischemia by aortic clamping and evaluated the protective effect of IPC, confirming the results presented here that this method may decrease the degree of lung injury. Another study that obtained similar results was that of Garbaisz et al.^[12]^, in which the authors also applied infrarenal aortic clamping as a method of ischemia and obtained pulmonary protection with IPC, although using times superior to those presented here for both ischemia (3h) and for reperfusion (4h). This shows that, for Wistar rats, 70 minutes of IR are sufficient to produce pulmonary lesions, and no further prolonged experiments are needed as the used by those authors.

Szijártó et al.^[13]^ also demonstrated IPC protection when IPC was performed on the lower limbs of rats, with three hours of ischemia and four hours of reperfusion. Santos et al.^[6]^ also performed mesenteric IR in rats, but used 30 minutes of ischemia and 60 minutes of reperfusion, causing intestinal lesions but not pulmonary lesions, thus demonstrating that these animals did not present significant damage to the lungs with these reduced periods of intestinal IR.

As mentioned, there are few publications aimed at evaluating remote lung damage against IR, especially using IPC as a protection method. Existing publications point to a promising role in this method, similar to other IR situations in which IPC provides tissue protection^[14]^. Despite this, there are few studies that have used IPC in clinical practice, which reinforces the importance of finding a pharmacological method that presents the same or greater efficacy, increasing interest in safe drugs such as statins and that at least their pleiotropic effect could be useful in such situations.

In the present study, pulmonary protection was obtained with the use of atorvastatin, in the same intensity as with IPC. As there are no studies with the same design used here, *i.e.* aortic clamping and atorvastatin use, the comparison with the literature is also impaired. Moreover, since the use of statins for the prevention of reperfusion injury is relatively new, the best route of administration and ideal dose are items to be better clarified in future research. Administration by gavage was chosen here with the intention of simulating what is practiced in humans, that is, the absorption by the gastrointestinal tract, aiming its clinical applicability.

Statins have been successfully tested for this purpose in several situations. Wu et al.^[15]^ performed renal IR in rats and demonstrated that atorvastatin decreased tissue injury in the control group. The same results were obtained by Cusumano et al.^[16]^ in renal IR of rats using atorvastatin.

Statins also protect other tissues in the presence of IR, such as heart^[17-19]^, nervous system^[20]^ and liver^[21]^. In the lung, the efficiency of statins was also demonstrated, as published by Matsuo et al.^[22]^, however, with a method different from the one used here, since these authors performed IR directly in the pulmonary hilum, thus not being a study of remote protection. In addition, these authors used rosuvastatin as a protective medicine and not atorvastatin as in the present research.

The mechanism of protection of statins to IR situations is due to its pleiotropic effect. By inhibiting a conversion of HMG-CoA to L-mevalonate, statins prevent a synthesis of isoprenoids, which are precursors of cholesterol biosynthesis, which serve as important lipid ligands for post-translational modification of intracellular proteins such as small GTPases, Rho, Rac and Ras. This protein isoprenylation allows a suitable subcellular localization and an intracellular circuit of proteins, which control various cellular functions, and an inhibition and pathways are important components of the pleiotropic effects of statins. The Rho pathway is related to oxidative stress, atherosclerosis and elevated blood pressure, signaling the pathway between the two crucial mechanisms, such as cytoskeletal remodeling and the ROS synthesis^[16]^.

In the development of this project, we did not know that the therapeutic methods applied would present the results shown here, so that an association group (IPC+A) was created aiming at enhancing tissue protection. However, there was no advantage in the association, since, in isolation, these therapeutic methods obtained mean tissue lesion statistically similar to the SHAM group, *i.e*., it would not be possible to have a lower lesion than was already achieved. Thus, it can be verified that atorvastatin has the capacity to protect the lungs in situations of reperfusion at a distance, at the same intensity as IPC, and it is possible to invest in research that confirms the best method of using these therapies to apply them in the clinical practice.

## CONCLUSION

IPC and atorvastatin were able to minimize lung reperfusion injury, alone or in combination.

**Table:** 

Authors' roles & responsibilities	
CHMS	Substantial contributions to the conception or design of the work; or the acquisition, analysis, or interpretation of data for the work; final approval of the version to be published
DMD	Substantial contributions to the conception or design of the work; or the acquisition, analysis, or interpretation of data for the work; final approval of the version to be published
BAKS	Substantial contributions to the conception or design of the work; or the acquisition, analysis, or interpretation of data for the work; final approval of the version to be published
HBDP	Substantial contributions to the conception or design of the work; or the acquisition, analysis, or interpretation of data for the work; final approval of the version to be published
EAN	Substantial contributions to the conception or design of the work; or the acquisition, analysis, or interpretation of data for the work; final approval of the version to be published
GSCV	Substantial contributions to the conception or design of the work; or the acquisition, analysis, or interpretation of data for the work; final approval of the version to be published
IOC	Substantial contributions to the conception or design of the work; or the acquisition, analysis, or interpretation of data for the work; final approval of the version to be published
JVCM	Substantial contributions to the conception or design of the work; or the acquisition, analysis, or interpretation of data for the work; final approval of the version to be published
JVDGO	Substantial contributions to the conception or design of the work; or the acquisition, analysis, or interpretation of data for the work; final approval of the version to be published
LESD	Substantial contributions to the conception or design of the work; or the acquisition, analysis, or interpretation of data for the work; final approval of the version to be published
MHMA	Substantial contributions to the conception or design of the work; or the acquisition, analysis, or interpretation of data for the work; final approval of the version to be published
TLS	Substantial contributions to the conception or design of the work; or the acquisition, analysis, or interpretation of data for the work; final approval of the version to be published
